# Mapping of Ionomic Traits in *Mimulus guttatus* Reveals Mo and Cd QTLs That Colocalize with *MOT1* Homologues

**DOI:** 10.1371/journal.pone.0030730

**Published:** 2012-01-24

**Authors:** David B. Lowry, Calvin C. Sheng, Zhirui Zhu, Thomas E. Juenger, Brett Lahner, David E. Salt, John H. Willis

**Affiliations:** 1 University Program in Genetics and Genomics, Duke University Medical Center, Durham, North Carolina, United States of America; 2 Department of Biology, Duke University, Durham, North Carolina, United States of America; 3 Section of Integrative Biology and Institute for Cellular and Molecular Biology, The University of Texas at Austin, Austin, Texas, United States of America; 4 Department of Horticulture and Landscape Architecture, Purdue University, West Lafayette, Indiana, United States of America; 5 Institute of Biological and Environmental Sciences, University of Aberdeen, Aberdeen, Scotland, United Kingdom; Lund University, Sweden

## Abstract

Natural variation in the regulation of the accumulation of mineral nutrients and trace elements in plant tissues is crucial to plant metabolism, development, and survival across different habitats. Studies of the genetic basis of natural variation in nutrient metabolism have been facilitated by the development of ionomics. Ionomics is a functional genomic approach for the identification of the genes and gene networks that regulate the elemental composition, or ionome, of an organism. In this study, we evaluated the genetic basis of divergence in elemental composition between an inland annual and a coastal perennial accession of *Mimulus guttatus* using a recombinant inbred line (RIL) mapping population. Out of 20 elements evaluated, Mo and Cd were the most divergent in accumulation between the two accessions and were highly genetically correlated in the RILs across two replicated experiments. We discovered two major quantitative trait loci (QTL) for Mo accumulation, the largest of which consistently colocalized with a QTL for Cd accumulation. Interestingly, both Mo QTLs also colocalized with the two *M. guttatus* homologues of *MOT1*, the only known plant transporter to be involved in natural variation in molybdate uptake.

## Introduction

Understanding natural genetic variation in the uptake, transport, and sequestration of mineral nutrients is crucial to determining how plants survive across different habitats. Since individual plants only grow in one location for the entirety of their lives they must be able to cope with different levels of available mineral nutrients. As a result, plants have evolved numerous mechanisms for regulation of the accumulation of nutrients. Natural variation in these processes appears to play an important role in plant growth [1]. Many of the mineral nutrient and trace elements accumulated by specific plant tissues also appear to be regulated in a coordinated fashion [2], [3]. With the coupling of high-throughput multi-element (ionomic) analytical techniques, such as Inductively Coupled Plasma-Mass Spectroscopy (ICP-MS) with genome-enabled genetics, it is now possible to efficiently study the genetic mechanisms involved in controlling these multiple element regulatory networks [4]–[6].

Studying genetic variation in elemental accumulation can provide major insights into how plants adapt to heterogeneity of soil types that comprise the natural landscape [7]–[9]. Further, adaptations to edaphic soil conditions can ultimately lead to evolution of reproductive isolating barriers and the formation of new species [10]–[12]. Studies of natural genetic variation have already led to the discovery of multiple genes involved in elemental accumulation in *Arabidopsis thaliana* [2], [13]–[16]. Knowledge of the mechanisms underlying natural variation in elemental accumulation will be crucial for the adaptation of cropping systems to provide food security under future global change scenarios [17], [18].

In this study, we examined the genetic architecture of natural variation in the accumulation of mineral elements in a cross between individuals from a coastal perennial and an inland annual population of the yellow monkeyflower, *Mimulus guttatus*. Overall, we quantified the accumulation of 20 elements including macronutrients (Ca, K, Mg, P, S), analogues of macronutrients (Rb, Sr), micronutrients (B, Co, Cu, Fe, Li, Mn, Mo, Ni, Se, Zn), and elements that can be harmful to plant growth (As, Cd, Na). Previous studies have identified multiple genomic regions associated with adaptive divergence in flowering time [19]–[22] and salt tolerance [23] between coastal perennial and inland annual ecotypes. Even so, little is known about metabolic variation among any populations of *M. guttatus*. The major goals of this study were to: 1) Characterize trait correlations and divergence in elemental accumulation between inland annual and coastal perennial accessions of *M. guttatus*, 2) Discover QTL associated with the variation in the elemental accumulation, and 3) Determine if these QTLs colocalize with candidate genes that could be the targets of follow up fine-mapping and molecular studies. Understanding the genetic architecture of natural variation in elemental accumulation between these coastal perennial and inland annual populations is an important first step in understanding potential metabolic adaptations within *M. guttatus* to divergent environmental conditions.

## Methods

### Mapping population

To discover QTLs involved in the leaf concentration of elements, we utilized a previously constructed recombinant inbred line (RIL) population. The RIL population was created from a cross between an outbred perennial individual from coastal sand dune habitat (DUN) in the Oregon Dune National Recreation Area (43°53′35″N 124°08′16″W) and IM62, an inbred line from an inland annual population (IM) located in montane habitat on Iron Mountain in the Oregon Cascade Mountains (44°24′03″N 122°08′57″W). The creation of this RIL mapping population is outlined by Hall & Willis [24]. Lowry et al. [23] subsequently constructed a genetic map for these RILs with 189 polymorphic genetic markers (details of markers can be found at www.mimulusevolution.org). The genotype data for the RIL population has been deposited in Dryad (www.datadryad.org, doi:10.5061/dryad.1500/1).

### Growth conditions

Ionomic analysis of RILs was conducted with a hydroponic experiment. RILs were germinated in Fafard 4P soil. Three replicates of 168 RILs were transplanted into 2.5 inch square pots filled with perlite a few days after germination. These pots were fully randomized across trays (32 pots per tray) and the trays were submerged in half-strength Hoagland's solution (pH = 6.0) such that the level of solution was 1 cm below the top surface of the perlite. Hoagland's solution was changed every 3–5 days to maintain similar nutrient concentrations throughout the experiment. To prevent plants from flowering, which could alter their physiology, they were grown under short days. All plants were grown for the entirety of their lives in a growth chamber at Duke University under 8 hour periods of light at 22°C and 16 hour periods of dark at 18°C with a light intensity of 225 umol/m^2^/s. Plant morphology of *M. guttatus* in these hydroponic conditions is comparable to the morphology when grown in soil in the same growth chambers. To confirm the results of the first experiment, we conducted a second experiment using the same conditions and experimental techniques. However, in this experiment we only grew a total of 142 RILs because of lower germination success and loss of lines to inbreeding depression. To assess the genetic divergence between DUN and IM for elemental accumulation, inbred parental lines (IM62 and DUN10) were also grown in each experiment. It should be noted that while IM62 is a parent of the RIL mapping population, DUN10 is not. The original DUN parent could not be maintained as an inbred seed line because it was outbred.

### Ionomic analysis

The second pair of true leaves from each replicate plant was collected with plastic forceps from plants 30 days after germination. Leaf tissue was briefly submerged in 0.05% Triton and rinsed in DI water. Leaves from all three replicates of each RIL were bulked into a single 15 mL plastic tube (VWR International) and dried in an oven at 90°C for 24 hours. Leaves were not pooled for parental inbred lines (Experiment 1: *N* = 10 IM and 10 DUN independent samples; Experiment 2: *N* = 16 IM and 16 DUN independent samples). Samples were then sent to the Purdue University ionomics laboratory (www.ionomicshub.org) for analysis of leaf elemental concentrations using inductively coupled plasma-mass spectroscopy (ICP-MS) [25]. About 10 mg of dried leaf tissue was weighed into 100×16 mm Pyrex tubes and digested with 1.05 mL of concentrated HNO_3_ (Mallinckrodt, AR Select grade) at 110°C for 4 hours. Each sample was diluted to 10.0 ml with 18 MΩ water and analyzed at N = 3 on a PerkinElmer Elan DRCe ICP-MS using a concentric nebulizer and cyclonic spray chamber (Glass Expansion) and a sample flow of 300 ul/min. Indium (EM Science, Gibbstown, New Jersey, USA) was used as an internal standard. National Institute of Standards and Technology traceable calibration standards were used for the calibration (ULTRAScientific, Kingstown, Rhode Island, USA). A total of 19 elements were quantified in the first experiment and 20 in the second, with As missing from the first experiment.

### General data analysis

The divergence between the IM62 and DUN10 lines was assessed using Wilcoxon Rank-Sum tests. Since, the RIL population was constructed using reciprocal crosses, it allowed for the assessment of cytoplasmic effects on ionomic concentration. Cytoplasmic effects across RILs were also examined with Wilcoxon Rank-Sum tests. To assess correlations between traits across RILs, pairwise Pearson product-moment correlation coefficients (r) were calculated for the ionomics traits based on the pooled sample point estimates for each RIL. Significance of parental inbred line divergence, cytoplasmic effects, and trait correlations was assessed using Bonferoni correction for multiple comparisons. To determine if results were replicable across experiments, data were analyzed separately for experiment one and two. All general data analysis was conducted in the statistical package JMP 8.02.

### QTL analysis

QTL mapping was conducted in R/qtl [26], which is a module of the statistical package R (www.r-project.org). Before QTL mapping, the effect of cytoplasm was removed by taking the residuals of a single factor ANOVA, where cytoplasm was a fixed effect. Normality was assessed for each phenotype with a Shapiro-Wilks normality test. Log transformations were conducted for phenotypes where normality was rejected.

We mapped QTL using the composite interval mapping procedure (CIM) implemented with Haley-Knott regression in R/qtl. Mapping was conducted at 1 cM steps across the genome with a maximum of three background covariate markers chosen using a forward stepwise procedure and a window size of 20 cM around each test position. The threshold of a maximum of three covariate markers was established through pilot interval mapping which identified only a few major effect QTLs. To establish alpha = 0.05 significant thresholds, the data was permutated 1000 times per each trait in each experiment. To establish a confidence limit for the position of each QTL we calculated the 1.5-LOD support interval around each significant QTL in R/qtl. Following QTL mapping, single marker analyses (ANOVA) were conducted with the marker closest to the peak of each significant QTL. The additive affect (2a) of each QTL was calculated as the difference between the means of the two alternative homozygous genotypes at the marker closest to the QTL peak. The percent variance explained was calculated as the *R^2^* of the model multiplied by 100.

To test for potential epistatic interactions between QTLs, we ran the scantwo function with the Haley-Knott regression in R/qtl on all ionomic traits. Penalties derived from 1000 permutations of scantwo were used to conduct stepwise QTL analysis. Here, multiple QTLs models were compared to identify the most likely QTL model. This analysis involved Haley-Knott regression and a maximum of three possible QTLs per model per trait. Epistasis was defined by the presence of a non-additive interaction of QTLs in the most likely stepwise model per each trait.

## Results

### Variation in leaf ion concentrations between accessions

**Figure 1:**
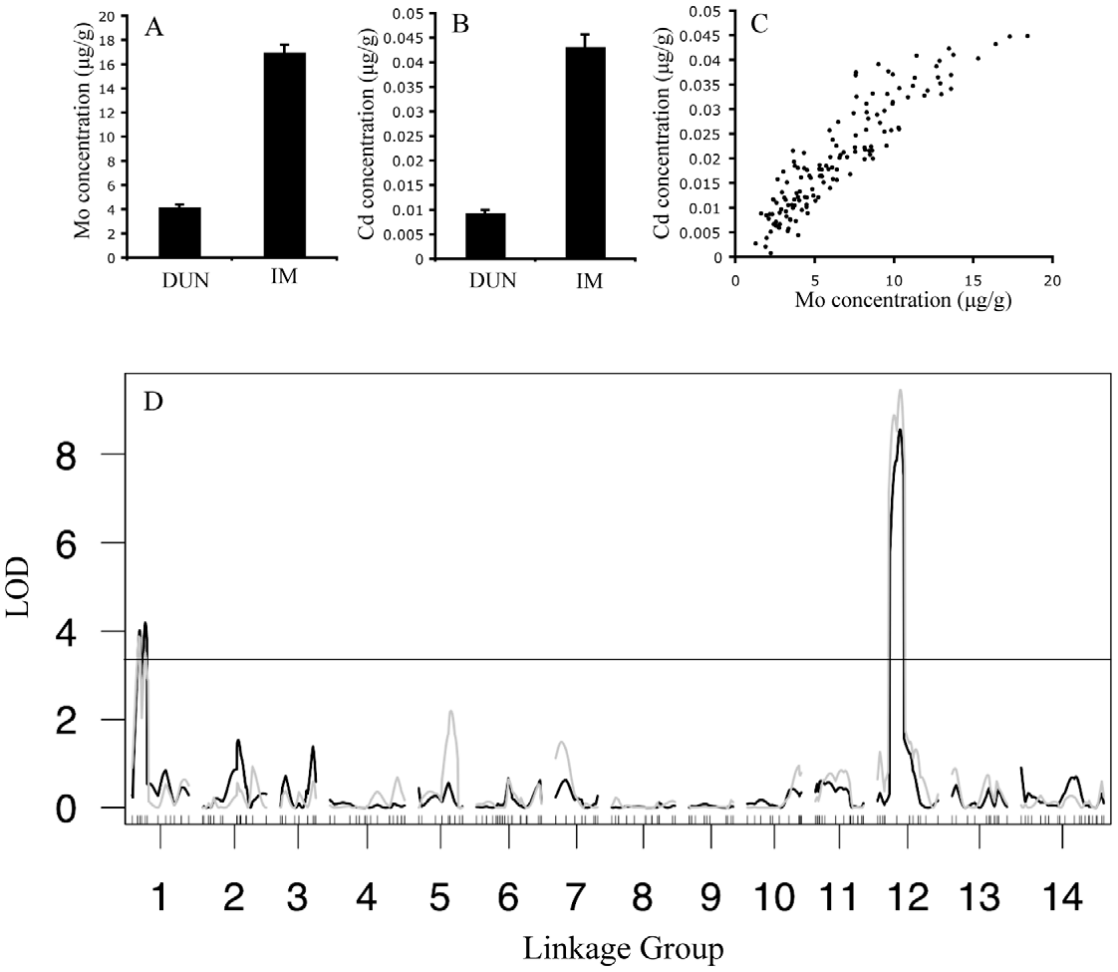
The relationship between Mo and Cd in the second experiment. Mean (+/−SE) of A) Mo and B) Cd leaf ion concentrations in DUN10 and IM62 lines. C) Strong genetic correlation between Mo and Cd in the RILs. D) QTL map shows overlap in the genetic architectures of Mo (black) and Cd (gray) leaf accumulation.

Overall, leaf elemental concentrations varied over six orders of magnitude, with Co, Cd, and Li having the lowest concentrations and Ca and K with the highest concentrations (µg g^−1^ dry weight). Four out of 19 ionomic traits significantly differed between IM62 and DUN10 in the first experiment, while nine out of 20 significantly differed in the second experiment (Table 1). Mo and Cd were the most strikingly divergent ions in both experiments, with IM62 leaves containing 4.35 times more Mo and 3.49 times more Cd than DUN10 leaves (Fig. 1).

**Table 1:** Parental means for elemental accumulation (µg g^−1^) in both experiments, and comparison by Wilcoxon Rank-Sum tests.

Trait	Exp	DUN mean	SE	IM mean	SE	*Z*	*P*-Value
Li	1	0.1068	0.0079	0.1446	0.0203	1.323	0.1859
B	1	26.75	0.59	31.47	1.81	2.306	0.0211
Na	1	1178	51	571	56	−3.742	0.0002*
Mg	1	1835	47	1980	54	1.701	0.0890
P	1	5988	255	7873	205	3.591	0.0003*
S	1	3052	119	3255	121	1.398	0.1620
K	1	57380	2440	50920	2510	−1.398	0.1620
Ca	1	12910	370	12750	260	−0.265	0.7913
Mn	1	37.42	2.81	42.77	1.56	1.021	0.3075
Fe	1	60.65	1.71	70.06	1.81	2.759	0.0058
Co	1	0.0187	0.0006	0.0183	0.0006	−0.265	0.7913
Ni	1	1.780	0.115	2.042	0.312	0.340	0.7337
Cu	1	10.37	0.95	11.64	1.03	0.567	0.5708
Zn	1	35.60	2.08	32.17	1.46	−1.398	0.1620
Se	1	0.0757	0.0085	0.0444	0.0045	−2.986	0.0028
Rb	1	9.961	0.393	8.740	0.421	−1.625	01041
Sr	1	40.74	2.34	40.39	1.95	−0.491	0.6232
Mo	1	3.26	0.21	15.15	0.68	3.742	0.0002*
Cd	1	0.0370	0.0021	0.0878	0.0028	3.742	0.0002*
Li	2	0.2878	0.0242	0.2357	0.0178	−1.677	0.0935
B	2	49.21	2.06	42.67	1.44	−2.356	0.0185
Na	2	3642	254	1810	209	−3.938	<0.0001*
Mg	2	2441	130	2558	45	1.187	0.2351
P	2	6761	198	9199	149	4.767	<0.0001*
S	2	4874	154	4012	74	−4.052	<0.0001*
K	2	72700	1210	61620	821	−4.353	<0.0001*
Ca	2	11650	610	13480	312	2.657	0.0079
Mn	2	95.78	4.77	77.45	1.40	−3.147	0.0016*
Fe	2	94.9	6.8	105.6	2.2	1.300	0.1935
Co	2	0.0383	0.0065	0.0396	0.0024	0.194	0.0523
Ni	2	0.3784	0.0460	0.3217	0.0223	−0.961	0.3365
Cu	2	11.08	0.44	13.00	0.50	2.431	0.0151
Zn	2	32.51	1.80	42.72	1.17	3.449	0.0006*
As	2	0.0279	0.0025	0.0173	0.0019	−3.147	0.0016*
Se	2	0.0797	0.0141	0.0505	0.0200	−1.150	0.2503
Rb	2	11.25	0.18	10.80	0.19	−1.866	0.0621
Sr	2	64.78	3.78	81.11	2.13	2.996	0.0027
Mo	2	4.198	0.191	16.98	0.63	4.805	<0.0001*
Cd	2	0.0094	0.0006	0.0432	0.0025	4.805	<0.0001*

*denotes traits that were significantly different after Bonferroni correction at alpha = 0.05.

Across the RILs, we detected significant effects of cytoplasm on leaf elemental concentration for four traits (B, Mg, P, K; Table S1) in the first experiment. However, there were no significant cytoplasmic effects for any element in the second experiment.

### Correlations between ionomic traits

We detected 48 significant trait correlations across RILs in the first experiment and 29 in the second experiment (Table 2). A total of 12 trait pairs were significantly correlated in both experiments, however not always in the same direction. The correlation between B and Sr reversed direction across experiments, being negatively correlated in the first experiment and positively correlated in the second. Elemental analogs, including Rb/K, Ca/Sr, and Na/Li, were strongly correlated in both experiments. One of the most striking positive correlations was between Mo and Cd, with average Pearson correlation coefficient of 0.83 across experiments (Fig. 1c).

**Table 2:** Pearson product-moment correlation coefficients (r) between leaf elemental concentration traits in the RILs.

	Li	B	Na	Mg	P	S	K	Ca	Mn	Fe	Co	Ni	Cu	Zn	As	Se	Rb	Sr	Mo	Cd
Li	**—**	0.33	**0.53**					**0.54**			**0.48**	0.40			0.59			0.53		
B		**—**			−0.31			0.30							0.36			**0.32**		
Na	**0.38**	−0.32	**—**																	
Mg		−0.42		**—**			−0.36	0.38		0.42								0.36		
P					**—**															
S	−0.29					**—**														
K		0.30					**—**		−0.56		−0.34						**0.71**			
Ca	**0.44**				−0.49	−0.32	−0.54	**—**			**0.41**				0.37		**−0.45**	**0.98**		
Mn		0.30	−0.34						**—**								−0.57			
Fe			−0.31							**—**										
Co	**0.49**				−0.48	−0.29	−0.54	**0.67**			**—**	**0.32**			0.54			**0.45**		
Ni					−0.40	−0.31	−0.33	0.53			**0.53**	**—**								
Cu									0.31				**—**							
Zn	−0.31				0.34								0.41	**—**						
As	NA	NA	NA	NA	NA	NA	NA	NA	NA	NA	NA	NA	NA	NA	**—**			0.45		
Se															NA	**—**				
Rb	−0.37				0.52		**0.83**	**−0.66**			−0.62	−0.42		0.34	NA		**—**	**−0.47**		
Sr		**−0.31**			−0.35		−0.62	**0.74**	−0.29		**0.53**	0.38	−0.35		NA		**−0.53**	**—**		
Mo		0.28	−0.30						0.30						NA				**—**	**0.92**
Cd									0.29		−0.31				NA				**0.73**	**—**

The lower diagonal list correlations for experiment 1 correlations and the upper diagonal for experiment 2.

Bold indicates traits significant in both experiments.

Only correlations that were significant after Bonferoni correction are reported in the table.

### Discovered QTLs

We discovered eight significant QTLs across seven ionomic traits in the first experiment and seven QTLs across four traits in the second experiment (Table 3). Only four QTLs were identified in both experiments. The four robust QTLs included a P QTL on linkage group 13, a Mo QTL on linkage group 1, and colocalizing Cd and Mo QTLs on linkage group 12 (Table 3; Fig. 1d). Out of the eight traits with significant QTLs, three of them (Mg, Ca, Mn) did not significantly differ between the IM and DUN parents in either experiment. Mn had two detected QTLs with opposing directional effects. Of the five traits that were significantly divergent between the parents in at least one experiment, three of them (Na, P, S) had QTLs that acted in the opposite direction of the parental divergence. Only the Mo/Cd QTLs on linkage groups 1 and 12 had effects in the same direction of the parental divergence, with the IM alleles contributing to greater uptake of both Mo and Cd than the DUN alleles (Fig. 1d). Across experiments, the linkage group 12 QTL explained 30–42% of the parental divergence in Mo and 30–31% of the parental divergence in Cd.

**Table 3:** Leaf mineral nutrient QTLs discovered by composite interval mapping.

Trait	Exp	Chr	Peak (cM)	Closest marker	1.5-LOD Interval	LOD	Threshold	2a	Variance explained
Na	1	14	134	e583	127–142	4.35	3.37	172.893	7.61
Mg	1	2	69	e582	62–76	3.99	3.52	−184.767	14.36
P	1	13	74	e547	69–78	7.74	3.26	−814.169	10.79
P	2	13	71	e747a	65–82	4.83	3.53	−555.914	8.26
S	1	12	69	e548	62–80	5.73	3.53	−588.413	14.76
Ca	1	8	12	e721b	2–20	5.22	3.56	−1355.61	17.54
Mn	2	3	48	e214	45–52	8.38	3.48	−12.995	25.15
Mn	2	12	61	e113	52–71	3.83	3.48	8.093	8.07
Mo	1	1	12	e387	4–29	3.73	3.35	2.741	10.21
Mo	1	12	42	dr1	35–47	15.75	3.35	4.998	37.52
Mo	2	1	18	e436	7–26	4.19	3.50	1.620	8.12
Mo	2	12	40	dr1	26–47	8.56	3.50	3.773	27.65
Cd	1	12	40	dr1	21–46	6.70	3.41	0.015	21.08
Cd	2	1	12	e387	7–37	3.88	3.40	0.006	8.95
Cd	2	12	40	dr1	22–48	9.45	3.40	0.011	25.75

The additive effect (2a) is positive for greater accumulation by the IM allele and negative for greater accumulation by the DUN allele.

None of the most likely stepwise QTL models included a significant interaction between QTLs. Thus, we found no evidence for epistasis for any of the ionomic traits.

### *MOT1* is a potential candidate gene underlying the natural variation in Mo

**Figure 2:**
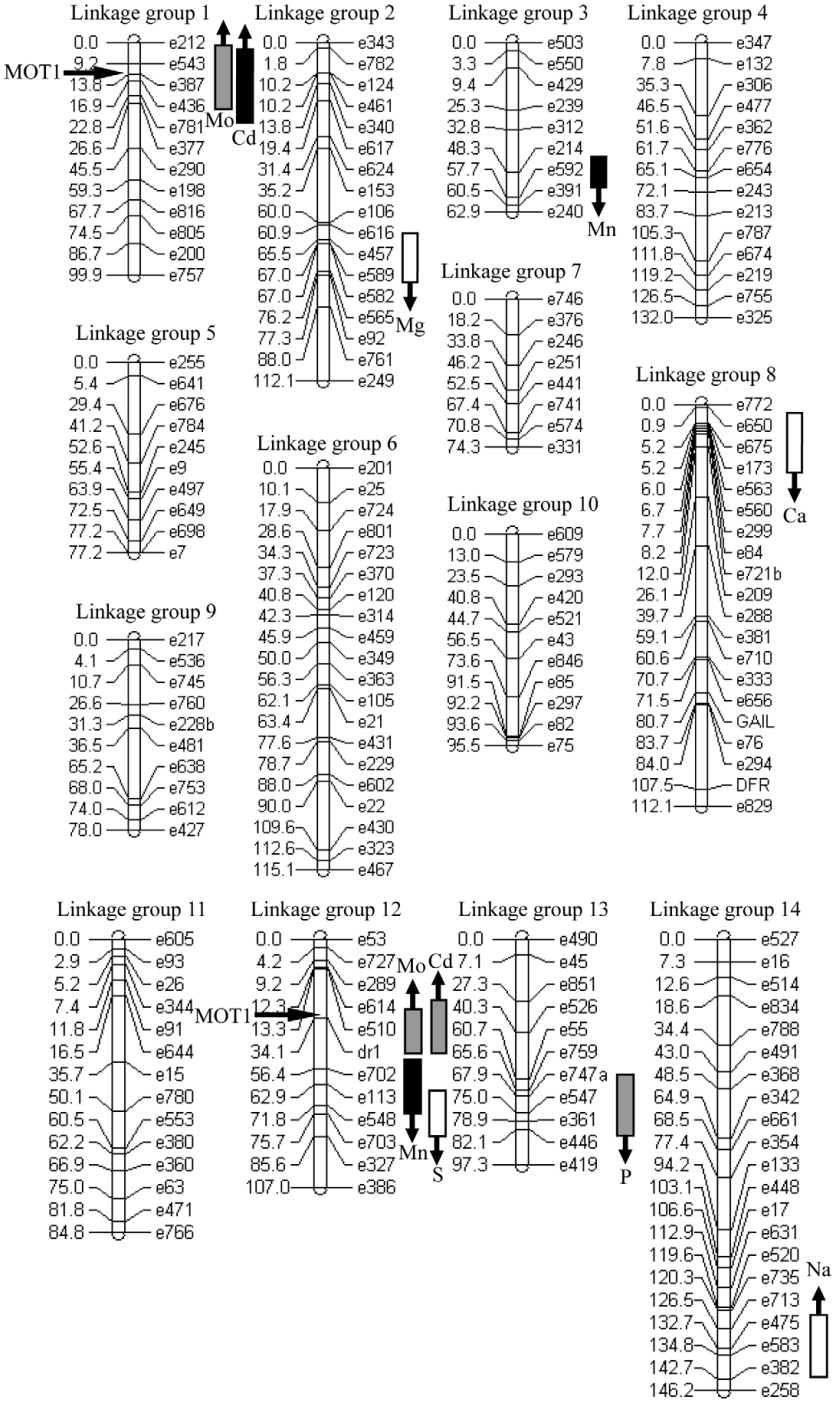
Locations of elemental accumulation QTLs. QTLs for experiment 1 (white), experiment 2 (black), and both experiments (gray) are plotted as 1.5 LOD intervals on the linkage map of the RILs. Locations of the two major homologues of *MOT1* are also plotted on the map. Up arrows indicate greater accumulation by the IM allele, while down arrows indicate greater accumulation by the DUN allele.

There are currently four plant proteins known to transport molybdate efficiently across cell and vacuolar membranes: *MOT1* from *Arabidopsis thaliana* and *Chlamydomonas reinhardtii*, *SHST1* from *Stylosanthes hamata*, *AtMOT2* from *A. thaliana* (a homologue of *MOT1*), and *CrMoT2* from *C. reinhardtii* (not a homologue of *MOT1*) [2], [27]–[34]: Because genome sequencing of the *M. guttatus* genome has been completed (www.phytozome.net/mimulus.php), we were able to use simple BLAST searches (tblastx) to determine if homologues of molybdate transporters co-localized with the Cd/Mo QTLs. In *Arabidopsis*, a deletion in the promoter of *AtMOT1* has been shown to play a major role in the natural variation in Mo accumulation among accessions [2]. There are two major BLAST hits for *AtMOT1* in *M. guttatus* (genome assembly 2.0), both with an e-value of 2.6e-124. Interestingly, one of the homologues is located within the 1.5-LOD interval of the large Mo/Cd QTL on linkage group 12 (Fig. 2). The second *MOT1* homologue maps to linkage group 1 within the 1.5-LOD interval of the Mo accumulation QTL detected in both experiments and the Cd accumulation QTL detected in experiment 2 (Table 3). Unlike *MOT1*, the other three molybdate transporters have not been linked to natural variation in Mo accumulation [29], [32]–[34]. The top five BLAST hits for *SHST1* did not colocalize with either of the Mo/Cd QTLs as they were located on linkage groups 14 (7e-152), 3 (4e-142), 2 (5e-134), 4 (2e-88), and 7 (4e-81). *AtMOT2* had its best hit on linkage group 4 (9e-141) with weaker hits to the two previously mentioned *MOT1* homologues on linkage groups 1 (8e-114) and 12 (1e-112). *CrMoT2* only had one significant hit in the *M. guttatus* genome on linkage group 6 (2e-51).

## Discussion

Our study is the first of its kind in *Mimulus* and expands the small diversity of plants where genetic architecture of the natural variation in the ionome has been explored. In this study, we were able to identify strong patterns of genetically based divergence in multiple ionomic traits, detect dozens of strong trait correlations, and discovered QTLs for eight of the 20 elemental abundances. In addition, we identified two major QTLs underlying a strong correlation between Cd and Mo, with each containing homologues of the candidate gene, *MOT1*.

### Genetic architecture of ionomic traits

Our analysis of genetic architecture failed to explain a large amount of the genetic divergence between IM and DUN or the elemental concentration trait correlations, despite the detection of QTLs for many traits. The DUN and IM lines compared in this experiment are genetically divergent in the accumulation of a number of elements. However, we identified few QTLs that had effects in the expected direction of these divergences. Likewise, there were strong and often consistent trait correlations across the RILs in both experiments, but we failed to identify many colocalizing QTLs across traits that could explain these correlations. As we have described in previous studies [20], [23], there are major reasons that our ability to detect QTLs were limited in this RIL mapping population. The major factors limiting QTL detection power included strong segregation distortion (48% of markers significantly distorted), phenotyping error, and a moderate level of missing genotype data (Mean ± SD, 17.85±11.83% per marker). Further, much of the natural genetic variation in ionomic traits could be controlled by a large number of loci that each had effects too small to detect given the sample size of this study. A recent study [3] detected a large number of small effect (*R^2^*<0.07) element accumulation QTLs in *A. thaliana* mapping populations. Such small effect QTLs were below the detection threshold of our study. Finally, it should be noted that the genetic variation underlying the divergence in elemental accumulation between DUN10 and IM62 might not be represented in the RILs since the IM62 line was a parent of the RILs while the DUN10 line was not.

### Natural genetic variation in Mo and Cd

The Mo/Cd QTLs on linkage group 1 and 12 are the only good candidates for locally adaptive loci identified in this study, as all other QTLs were only detected in one of the two experiments or had effects that were in the opposite direction of the parental divergence of the traits. Mo was the most divergent element measured between the DUN10 and IM62 accessions. This finding is consistent with a recent study in *A. thaliana*, where Mo was the most variable ion with significant divergence in 44 pairwise comparisons among 12 accessions [3]. Because this is the first exploration of natural genetic variation in *Mimulus*, it is unknown how variable Mo is within and among populations. Further studies should examine whether there is a consistent divergence in Mo that correlates with environmental variation, such as the perennial/annual ecotype division in *Mimulus* or particular soil types [21], [35], [36].

Mo is one of 14 essential plant elements and plays a key role as a cofactor component at the catalytic center of a diverse array of enzymes [27], [31], [37], [38]. Mo deficiency has been observed in many crop species [27], [39], [40], but Mo toxicity is rare. Mo deficiency can lead to poor nitrogen health and is often misdiagnosed as a deficiency of another plant nutrient [27], [30]. Because of the very low levels of accumulation of Mo in plants, its potential role in adaptation has been difficult to study until recently [2]. In contrast to Mo, Cd is toxic and non-essential to most plants, with the exception being rare Cd hyperaccumulators [41]–[44]. Cd is thought to enter the root primarily through essential metal uptake systems, especially Ca, Fe, Mn, and Zn transporters [44], [45].

The exact mechanisms of uptake and accumulation of molybdenum in plants are unclear (reviewed in [31]). However, recent studies have identified two homologous transporters of molybdate (*AtMOT1*, *AtMOT2*) in *A thaliana* [2], [28], [30], [34]. This appears to be a common mechanism of Mo metabolism across plants as a *MOT1* homologue has also been found to control accumulation of Mo in the green algae *Chlamydomonas reinhardtii* [32]. One possible explanation of how Cd and Mo accumulation could be connected through *MOT1* is that both elements are tightly linked through Fe-homoeostasis in *A. thaliana*. Under Fe-deficiency and constitutively in the Fe-deficient mutant *frd3*, Cd is increased while Mo decreases [4]. Reduction of Mo may be due to acidification of the rhizosphere during Fe-deficiency, which would result in Mo becoming less bioavailable and/or as a result of down regulation of *MOT1*. Whereas, increased Cd is likely due to increased activity of the Fe-transporter *IRT1* [46]. In the *A. thaliana* mutant *mot1-1* there is an 80% reduction in Mo with no effect on Cd [2].

In contrast to *A. thaliana*, there is a strong positive relationship between Mo and Cd in *M. guttatus*. Molybdopterin and FeS cluster biosynthesis are linked in the mitochondria through *ATM3*, and Fe-homeostasis in *atm3* appears to be perturbed, with elevated *IRT1* expression and reduced ferritin [47]. Perhaps in *Mimulus* increased activity of *MOT1* is associated with increased expression of *IRT1*. Another possibility is that *MOT1* may interact with other transporters that play a key role in Cd accumulation, such as *HMA3* in *A. thaliana* [45]. Alternatively, the colocalization of the Mo and Cd QTLs could simply be due to tight linkage of causative genes. Further fine-mapping and molecular analysis will be necessary to resolve whether natural variation in the *MOT1* homologues is the cause of the large Mo QTL and whether there is a direct molecular connection between Mo and Cd accumulation.

## Supporting Information

Table S1 Ionomic traits with significant cytoplasmic effects in the RILs after Bonferroni correction.
(DOCX)

## References

[1] Prinzenberg AE, Barbier H, Salt DE, Stich B, Reymond M (2010). Relationships between Growth, Growth Response to Nutrient Supply, and Ion Content Using a Recombinant Inbred Line Population in *Arabidopsis*.. Plant Physiology.

[2] Baxter I, Muthukumar B, Park HC, Buchner P, Lahner B (2008). Variation in molybdenum content across broadly distributed populations of *Arabidopsis thaliana* is controlled by a mitochondrial molybdenum transporter (*MOT1*).. PLoS Genetics.

[3] Buescher E, Achberger T, Amusan I, Giannini A, Ochsenfeld C (2010). Natural genetic variation in selected populations of *Arabidopsis thaliana* is associated with ionomic differences.. PLoS One.

[4] Baxter I (2009). Ionomics: studying the social network of mineral nutrients.. Current Opinion in Plant Biology.

[5] Tian H, Baxter IR, Lahner B, Reinders A, Salt DE (2010). *Arabidopsis* NPCC6/NaKR1 is a phloem mobile metal binding protein necessary for phloem function and root meristem maintenance.. Plant Cell.

[6] Chao DY, Gable K, Chen M, Baxter I, Dietrich CR (2011). Sphingolipids in the root play an important role in regulating the leaf ionome in *Arabidopsis thaliana*.. Plant Cell.

[7] Antonovics, Bradshaw AD (1970). Evolution in closely adjacent plant populations. VIII. Clinal patterns at a mine boundary.. Heredity.

[8] Baxter I, Brazelton JN, Yu DN, Huang YS, Lahner B (2010). A coastal cline in Sodium accumulation in *Arabidopsis thaliana* is driven by natural variation of the sodium transporter *AtHKT1;1*.. PLoS Genetics.

[9] Harrison S, Rajakaruna N (2011). Serpentine: The evolution and ecology of a model system.

[10] Macnair MR, Christie P (1983). Reproductive isolation as a pleitropic effect of copper tolerance in *Mimulus guttatus*?. Heredity.

[11] Gardner M, Macnair M (2000). Factors affecting the co-existence of the serpentine endemic *Mimulus nudatus* Curran and its presumed progenitor, *Mimulus guttatus* Fischer ex DC.. Biological Journal of the Linnean Society.

[12] Kay KM, Ward KL, Watt LR, Schemske DW, Harrison S, Rajakaruna N (2011). Plant Speciation.. Serpentine: The evolution and ecology of a model system.

[13] Rus A, Baxter I, Muthukumar B, Gustin J, Lahner B (2006). Natural variants of *AtHKT1* enhance Na+ accumulation in two wild populations of *Arabidopsis*.. PLoS Genetics.

[14] Loudet O, Saliba-Colombani V, Camilleri C, Calenge F, Gaudon V (2007). Natural variation for sulfate content in *Arabidopsis thaliana* is highly controlled by APR2.. Nature Genetics.

[15] Morrissey J, Baxter IR, Lee J, Li LT, Lahner B (2009). The ferroportin metal efflux proteins function in iron and cobalt homeostasis in *Arabidopsis*.. Plant Cell.

[16] Kobayashi Y, Kuroda K, Kimura K, Southron-Francis JL, Furuzawa A (2008). Amino acid polymorphisms in strictly conserved domains of a P-type ATPase HMA5 are involved in the mechanism of copper tolerance variation in *Arabidopsis*.. Plant Physiology.

[17] Lobell DB, Burke MB, Tebaldi C, Mastrandrea MD, Falcon WP (2008). Prioritizing climate change adaptation needs for food security in 2030.. Science.

[18] Ingram JSI, Gregory PJ, Izac AM (2008). The role of agronomic research in climate change and food security policy.. Agriculture, Ecosystem & Environment.

[19] Hall MC, Basten CJ, Willis JH (2006). Pleiotropic quantitative trait loci contribute to population divergence in traits associated with life-history variation in *Mimulus guttatus*.. Genetics.

[20] Hall MC, Lowry DB, Willis JH (2010). Is local adaptation in *Mimulus guttatus* caused by trade-offs at individual loci?. Molecular Ecology.

[21] Lowry DB, Rockwood RC, Willis JH (2008). Ecological reproductive isolation of coast and inland races of *Mimulus guttatus*.. Evolution.

[22] Lowry DB (2010). Landscape evolutionary genomics.. Biology Letter.

[23] Lowry DB, Hall MC, Salt DE, Willis JH (2009). Genetic and physiological basis of adaptive salt tolerance divergence between coastal and inland *Mimulus guttatus*.. New Phytologist.

[24] Hall MC, Willis JH (2006). Divergent selection on flowering time contributes to local adaptation in *Mimulus guttatus* populations.. Evolution.

[25] Baxter I, Ouzzani M, Orcun S, Kennedy B, Jandhyala SS (2007). Purdue Ionomics Information Management System. An integrated functional genomics platform.. Plant Physiology.

[26] Broman KW, Wu H, Sen S, Churchill GA (2003). R/qtl: QTL mapping in experimental crosses.. Bioinformatics.

[27] Kaiser BN, Gridley KL, Brady JN, Phillips T, Tyerman SD (2005). The role of molybdenum in agricultural plant production.. Annals of Botany.

[28] Tomatsu H, Takano J, Takahashi H, Watanabe-Takahashi A, Shibagaki N (2007). An *Arabidopsis thaliana* high-affinity molybdate transporter required for efficient uptake of molybdate from soil.. Proceedings of the National Academy of Sciences U S A.

[29] Fitzpatrick KL, Tyerman SD, Kaiser BN (2008). Molybdate transport through the plant sulfate transporter *SHST1*.. FEBS Letters.

[30] Ide Y, Kusano M, Oikawa A, Fukushima A, Tomatsu H (2011). Effects of molybdenum deficiency and defects in molybdate transporter *MOT1* on transcript accumulation and nitrogen/sulphur metabolism in *Arabidopsis thaliana*.. Journal of Experimental Botany.

[31] Mendel RR, Schwarz G (2011). Molybdenum cofactor biosynthesis in plants and humans.. Coordination Chemistry Reviews.

[32] Tejada-Jimenez M, Llamas A, Sanz-Luque E, Galvan A, Fernandez E (2007). A high-affinity molybdate transporter in eukaryotes.. Proceedings of the National Academy of Sciences U S A.

[33] Tejada-Jimenez M, Galvan A, Fernandez E (2011). Algae and humans share a molybdate transporter.. Proceedings of the National Academy of Sciences U S A.

[34] Gasber A, Klaumann S, Trentmann O, Trampczynsk A, Clemens (2011). Identification of an *Arabidopsis* solute carrier critical for intracellular transport and inter-organ allocation of molybdate.. Plant Biology.

[35] Wu CA, Lowry DB, Nutter LI, Willis JH (2010). Natural variation for drought-response traits in the *Mimulus guttatus* species complex.. Oecologia.

[36] Lowry DB, Willis JH (2010). A widespread chromosomal inversion polymorphism contributes to a major life-history transition, local adaptation, and reproductive isolation.. PLoS Biology.

[37] Bittner F, Mendel RR (2010). Cell biology of molybdenum.. Plant Cell Monographs.

[38] Schwarz G, Mendel RR, Ribbe MW (2009). Molybdenum cofactors, enzymes and pathways.. Nature.

[39] Hewitt EJ (1956). Symptoms of molybdenum deficiency in plants.. Soil Science.

[40] Gupta UC (1997). Molybdenum in agriculture.

[41] di Toppi LS, Gabbrielli R (1999). Response to cadmium in higher plants.. Environmental and Experimental Botany.

[42] Jiang RF, Ma DY, Zhao FJ, McGrath SP (2005). Cadmium hyperaccumulation protects *Thlaspi caerulescens* from leaf feeding damage by thrips (*Frankliniella occidentalis*).. New Phytologist.

[43] Poschenrieder C, Tolra R, Barcelo J (2006). Can metals defend plants against biotic stress?. Trends in Plant Science.

[44] Verbruggen N, Hermans C, Schat H (2009). Molecular mechanisms of metal hyperaccumulation in plants.. New Phytologist.

[45] Verbruggen N, Hermans C, Schat H (2009). Mechanisms to cope with arsenic or cadmium excess in plants.. Current Opinion in Plant Biology.

[46] Vert G, Grotz N, Dedaldechamp F, Gaymard F, Guerinot ML (2002). *IRT1*, an *Arabidopsis* transporter essential for iron uptake from the soil and for plant growth.. Plant Cell.

[47] Bernard DG, Cheng YF, Zhao YD, Balk J (2009). An Allelic Mutant Series of ATM3 Reveals Its Key Role in the Biogenesis of Cytosolic Iron-Sulfur Proteins in *Arabidopsis*.. Plant Physiology.

