# Supplementary material for: Mapping of Ionomic Traits in Mimulus guttatus Reveals Mo and Cd QTLs That Colocalize with MOT1 Homologues
Source: PLoS One. 2012 Jan 24;7(1):e30730. doi: 10.1371/journal.pone.0030730 (PMC3265502; doi:10.1371/journal.pone.0030730)
Supplement: Table S1 — Ionomic traits with significant cytoplasmic effects in the RILs after Bonferroni correction. (DOCX) [file pone.0030730.s001.docx]

**Table S1: Ionomic traits with significant cytoplasmic effects in the RILs after Bonferroni correction.**

| Trait | Exp | DF | DUN cytoplasm | SE | IM cytoplasm | SE | *Z* | *P*-value |
| --- | --- | --- | --- | --- | --- | --- | --- | --- |
| B | 1 | 167 | 25.51 | 0.42 | 28.59 | 0.57 | 4.150 | <0.0001 |
| Mg | 1 | 167 | 1656 | 23 | 1475 | 39 | -4.833 | <0.0001 |
| P | 1 | 167 | 7191 | 100 | 6628 | 119 | -3.967 | <0.0001 |
| K | 1 | 167 | 55740 | 1000 | 61740 | 1618 | 3.198 | 0.0014 |
